# Identification of a Novel Serum Biomarker for Tuberculosis Infection in Chinese HIV Patients by iTRAQ-Based Quantitative Proteomics

**DOI:** 10.3389/fmicb.2018.00330

**Published:** 2018-02-26

**Authors:** Cong Chen, Tao Yan, Liguo Liu, Jianmin Wang, Qi Jin

**Affiliations:** ^1^MOH Key Laboratory of Systems Biology of Pathogens, Institute of Pathogen Biology, Chinese Academy of Medical Sciences and Peking Union Medical College, Beijing, China; ^2^Collaborative Innovation Center for Diagnosis and Treatment of Infectious Diseases, Hangzhou, China

**Keywords:** HIV, tuberculosis, biomarkers, proteomics, iTRAQ-based proteomics

## Abstract

Tuberculosis (TB) is a major comorbidity in HIV patients as well as a serious co-epidemic. Traditional detection methods are not effective or sensitive for the detection of *Mycobacterium tuberculosis* at the early stage. TB has become a major cause of lethal on HIV patients. We employed isobaric tags for relative and absolute quantitation (iTRAQ) technology to identify the different host responses in HIV-noTB and HIV-TB patients’ sera. Given the diversity of HIV subtypes, which results in a variety of host responses in different human populations, we focused on the Chinese patients. Of the 25 proteins identified, 7 were increased and 18 were decreased in HIV-TB co-infected patients. These proteins were found to be involved in host immune response processes. We identified a candidate protein, endoglin (ENG), which showed an 4.9 times increase by iTRAQ and 11.5 times increase by ELISA. ENG demonstrated the diagnostic efficacy and presented a novel molecular biomarker for TB in HIV-infected Chinese patients. This study provides new insight into the challenges in the diagnosis and effective management of patients with HIV-TB.

## Introduction

Tuberculosis (TB), a bacterial infectious disease, is a major comorbidity in HIV-infected individuals, many of whom suffer from acquired immunodeficiency syndrome (AIDS) (Harries, 1990; Getahun et al., 2010; Hickey et al., 2015; Ai et al., 2016). The World Health Organization reports that a third of HIV-infected individuals in the world are co-infected with *Mycobacterium* species, mainly *Mycobacterium tuberculosis* (Mtb), which causes a resurgence of tuberculosis with the HIV pandemic (Andrews et al., 2012). Approximately 10% of individuals with latent Mtb infection eventually develop TB in their lifetime. It is well known that mycobacterial co-infection can promote the progression of AIDS in HIV patients. In addition, studies have revealed that mycobacteria can more easily infect individuals with HIV infection than those (Pedersen et al., 1997; Corbett et al., 2003). These findings suggest a mutualistic relationship between the two pathogens. Given that mycobacteria exist widely in the environment, such as in food, soil, water and air, HIV patients have a high risk of exposure to Mtb. Furthermore, management and treatment of mycobacterial infections in HIV patients is difficult due to drug toxicity, drug interactions, and TB-related immune reconstitution inflammatory syndrome (von Reyn et al., 2011; Narasimhan et al., 2013). In general, TB in HIV-infected patients is a major issue in the field of infectious diseases.

Prevention and reduction of transmission are the key strategies for improving the control of TB, which requires sensitive diagnoses at the early stages of the disease (Dheda et al., 2017). However, this is the most challenging issue, because specimens for the detection of Mtb are not always readily obtainable. Additionally, it would take several weeks for sputum culture, but the results are not sensitive. Therefore, non-invasive biomarkers with high sensitivity, specificity, and reproducibility are important for the early diagnosis of TB. Lack of effective biomarkers has led researchers to develop novel technologies to discover more sensitive biomarkers for the detection of TB. Given the threat of co-infection with HIV and Mtb, identification of effective biomarkers for early diagnosis of TB is an urgent need.

Proteomics-based technology for detecting biomarkers in serum is an effective method for the diagnosis of TB (Song et al., 2014; Xu et al., 2014; Achkar et al., 2015). In contrast, traditional methods of detecting TB using antibodies are limited by the lack of sensitivity to the great variety of TB antigens. Instead of antibodies to detect each individual TB antigen, proteomics allows the analysis of all proteins in the serum. In addition, the change in the levels of TB-associated proteins in patient serum can be detected by proteomics. In terms of the human host response to TB, determination of changes in the levels of protein biomarkers by proteomics does not depend on the detection of Mtb. Sputum samples are not required for proteomics methods. A previous study using a combined method of mass spectrometry and magnetic beads identified TB fibrinogen as a potential biomarker in the serum (Liu et al., 2013, 2015). Another study applied surface-enhanced laser desorption ionization time of flight mass spectrometry (SELDI-TOF-MS) and protein-chip technology to distinguish proteins from TB patients and control (Arthur and Wilkins, 2004; Zhang et al., 2012). Recent studies identified TB biomarker panels from patient sera by iTRAQ-based proteomics analysis of protein levels between samples from TB patients and control individuals (with either no or latent *M. tuberculosis* infection) (Yang et al., 2015; Wang et al., 2016). The results showed that the differentially expressed proteins were involved in immune response, lipid metabolism and tissue repair. Due to the mutualism in the pathogenesis of HIV and TB, human host responses against TB in HIV patients could offer an opportunity for sensitive detection of TB in the serum using proteomics. In these studies, TB-uninfected HIV individuals expressed proteins in response to TB that were different from those expressed in TB and HIV co-infected patients. These data indicate that host protein biomarkers may be useful in testing for TB in serum. However, the patients in those studies were from African, Asian, and South and Central American countries, with a large proportion of them born outside of those countries. In addition, owing to the limited size of total samples, there was a lack of racial representation.

It was well known that host responses to HIV differ considerably between different populations. The HIV transmission process varies because of the diversity of viral subtypes and human host responses. Additionally, differences in viral subtypes result in variable rates of disease progression. For example, hosts carrying specific HLA class I types exhibit different HIV-1 transmission and disease progression (Taylor et al., 2008; Hemelaar, 2012). Thus, studies focusing on populations of a specific race might provide more insights into biomarkers for testing for the presence of TB in HIV-infected patients. There is a predominant HIV-1 subtype in China that recent studies have shown is different from those found in Western and African countries (Lu et al., 2008; Liao et al., 2010; Su et al., 2014). In this study, we focused on Chinese HIV patients who are co-infected with TB. Traditional detection methods, such as sputum culture, and current molecular diagnostic approaches are not effective or sensitive for the detection of Mtb at the early stage when prevention and treatment of TB are required. Here, we used iTRAQ-based proteomics to determine protein levels in blood. By comparing HIV-infected patients and HIV-TB co-infected patients, we identified 25 proteins, including 7 were increased and 18 were decreased in HIV-TB co-infected patients that indicated a set of host response proteins as candidate biomarkers for the detection of TB in HIV patients in China. Next, we tested 52 patients serum samples by using ELISA. We confirmed that expression of endoglin (ENG) significantly increased by more than ten times, and could serve as a molecular biomarker for TB in HIV-infected patients. The results broaden the scope of TB biomarkers.

## Materials and Methods

### Ethics Statement

The use of human serum samples was reviewed and approved by the Ethics Committee of the Institute of Pathogen Biology at the Chinese Academy of Medical Sciences and Peking Union Medical College. TB infection was diagnosed when the sputum smear was positive and/or sputum culture result was positive and/or PCR result was positive or when one or more positive results were obtained. HIV infection was laboratory confirmed by reverse-transcription polymerase chain reaction. 16 patients (including 8 were co-infected with TB) with infection by HIV-1 B subtype, which is the predominant genotype in China, were selected in this study. Serum samples were collected after written informed consent was obtained. All experiments were performed according to the standard operating procedures of biosafety level 2 facilities.

### Sample Collection and Protein Extraction

**FIGURE 1:**
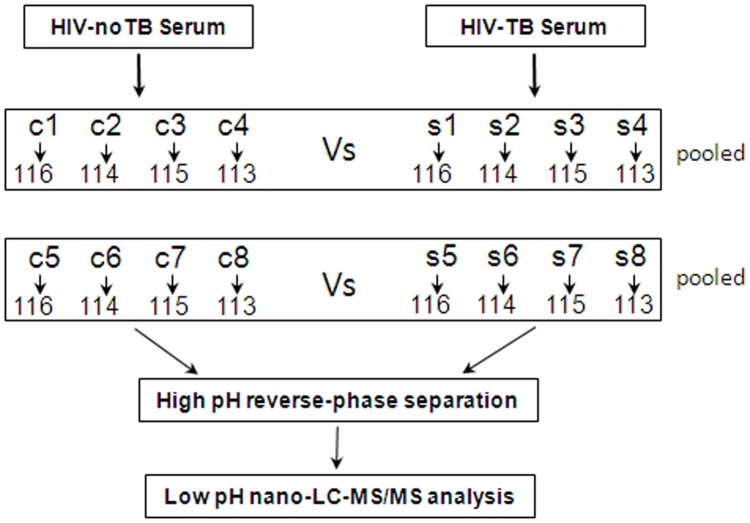
Schematic outline of sample preparation and iTRAQ analysis of sera from HIV-noTB and HIV-TB co-infected patients.

For 2D-LC-MS/MS, serum samples from 16 Chinese patients were collected and divided into two groups: HIV-noTB and HIV-TB co-infection. For ELISA assay, serum samples were collected, including 33 HIV-infected and 35 HIV-TB co-infected. All serum samples were processed following previously published protocols to minimize pre-analytical variation (Vasudev et al., 2008). In brief, the blood samples were allowed to clot for 1 h at 37°C; the sera were further centrifuged at 3000 *g*/min for 20 min at 4°C, and then the supernatant was transferred to a new tube. Serum sample from each individual was frozen in aliquots at -80°C until further testing. The workflow of the whole study is shown in **Figure 1**.

Proteins were extracted from the samples using ProteoMiner Protein Enrichment Kits (Bio-Rad Laboratories Inc., United States) following the manufacturer’s protocol. In brief, the serum samples from patients were sorted into two groups: HIV-noTB and HIV-TB co-infection. Equal volumes of serum samples from each group were mixed for iTRAQ analysis. The mixed serum samples from the two groups were subjected to high abundance protein depletion. The protein concentration was determined by BCA assay (TransGen Biotech, Beijing, China).

### iTRAQ Labeling

Ten microgram proteins from each sample were precipitated with ice-cold acetone, reconstituted in 100 μl TEAB with 100 mM stock solution and then digested with Trypsin Gold (Promega, Madison, WI, United States) at a certain ratio. Then, the resultant peptide mixtures were labeled with chemicals according to the manufacturer’s protocol in preparation for 8-plex iTRAQ (AB SCIEX, United States). The peptides were labeled with isobaric tags and incubated at room temperature for 2 h. The isobaric tag-labeled samples were pooled. Subsequently, equal amounts of labeled samples were desalted with Sep-Pak Vac V18 cartridges (Waters Corporation, United States) and dried by vacuum centrifugation for further usage.

### 2D-LC-MS/MS

The combined mixtures were reconstituted in buffer A (20 mM ammonium formate in water, pH 10.0). Later, the solutions were fractionated with a reverse-phase column (XBridge C18, 3.5 μm, 2.1 mm × 50 mm, Waters Corporation, United States) to obtain high pH separation (ACQUITY UPLC system, Waters Corporation, Milford, MA, United States). High pH separation was carried out with a linear gradient of 5 – 35% buffer B (20 mM ammonium formate in 90% ACN, pH 10.0). The column flow was set at a constant rate of 200 μl/min at room temperature. After separation, the column was re-equilibrated to initial conditions for 15 min. Finally, 20 fractions were collected and dried by vacuum centrifugation for further usage.

The above fractions were then analyzed using a Nano-ACQUITY UPLC system (Waters Corporation, United States) connected to a quadrupole-orbitrap mass spectrometer (Q-Exactive; Thermo Fisher Scientific, Bremen, Germany). The main procedures were as follows: re-suspension of the samples with 32 μl buffer C (0.1% formic acid), separation by nano-LC and analysis by online electrospray tandem mass spectrometry. Subsequently, the peptide sample (8 μl) was fractionated by a trap column (Thermo Scientific Acclaim PepMap C18, 100 μm × 2 cm) at a constant rate of 10 μl/min and then subjected to an analytical column (Acclaim PepMap C18, 75 μm × 50 cm) with a linear gradient of 2–40% buffer D (0.1% formic acid in ACN) at a constant rate of 300 nl/min at 40°C. After nano-LC separation, the column was re-equilibrated at the initial conditions for 15 min. The inlet of the mass spectrometer was adjusted to have an electrospray voltage of 2.2 kV. Mass spectrometry was performed on a data-dependent mode to automatically switch between MS and MS/MS acquisition. Full-scan survey MS spectra (350 – 1200 m/z) were obtained at a resolution of 70 K, followed by supplemental high-energy collisional dissociation (HCD) MS/MS scans at a resolution of 17.5 K. Under these conditions, one microscan was acquired with a dynamic exclusion of 30 s.

### Statistical Analysis of the iTRAQ Data

For protein identification, we carried out the following procedure. First, we transferred the raw files into .mzXML and .mgf formats with an MS convert module in Trans-Proteomic Pipeline (TPP 4.6.2). Second, we used Proteome Discoverer software version 1.4 from Thermo Scientific with MASCOT (version 2.3.0, Matrix Science, London, United Kingdom) to search for proteins against a human database (The Universal Protein Resource^1^; released on 2014-04-10, with 20,264 entries). The search parameters were specific trypsinization, peptide mass tolerance of 10 parts per million (ppm), fragment ion mass tolerance of 0.05 Da, fixed modifications such as carbamidomethyl (C) and iTRAQ 8-plex (N-term), and variable modifications such as oxidation (M) and iTRAQ 8-plex (T). To validate the identification quality, we performed analysis using Scaffold software (version Scaffold_4.3.2, Proteome Software Inc., Portland, OR, United States). To reduce the probability of false peptide identification, only those with significance scores of 99% confidence interval greater than their “identity” by Mascot probability analysis and false discovery rate (FDR) of <1% were considered positive (**Supplementary Table S1**). The raw data in this study has been deposited to the iProX database with identifier IPX0001146000.

### ELISA

To further confirm the alterations in the expression patterns of selected proteins in the serum of the experimental groups, ELISA was performed. The concentrations of ENG (CUSABIO, CSB-E10030h, China), PSMB2 (CUSABIO, CSB-E17836h, China), HSP90AA1 (CUSABIO, CSB-E13462h, China), HSPA8 (CUSABIO, CSB-EL010829HU, China), CHI3L1 (CUSABIO, CSB-E13608h, China), CAP1 (CUSABIO, CSB-EL004486HU, China), ORM1 (CUSABIO, CSB-EL017237HU, China), RETN (CUSABIO, CSB-E06884h, China), LPA (CUSABIO, CSB-EQ028005HU, China) and ZYX (CUSABIO, CSB-EL027165HU, China) in the serum samples were quantified using ELISA according to the manufacturer’s instructions. All the procedures were performed at 37°C. In brief, a 96-well plate was added with 100 μl of standard or sample to each well and then incubated 2 h. Both standards and samples were done in duplicate. Subsequently, remove the liquid of each well, added 100 μl HRP-avidin into the wells, and continued to incubate for 1 h. After washing, 90 μl of TMB substrate was added to all wells and incubated 15 – 30 min. Finally, 50 μl of stop solution was added to each well and the plate was read at 450 nm on an ELISA plate reader (Tecan) within 5 min. The data was exported to Excel, replicates were averaged and the standard dilutions were fit to a linear curve.

### Gene Ontology (GO) Analysis

For the detection of differentially expressed proteins, the quantitative protein ratio needed to be higher than 2 for up-regulation or lower than 0.5 for down-regulation. Only ratios with *p*-values < 0.05 were considered significant by paired *t*-test. Functional annotations of proteins were analyzed by GO annotation and pathway enrichment analysis using the online tool DAVID (Database for Annotation, Visualization and Integrated Discovery^2^). GO analysis regarding the functional annotation of protein encompassed three aspects: biological processes, molecular functions, and cell components. Protein–protein interaction (PPI) networks were constructed using the STRING webtool^3^.

### Statistical Analysis

Statistical analysis was performed with Statistical Program for Social Sciences (SPSS) (SPSS Inc., version 19.0, United States). The data were reported as the means ± SD, and the quantitative variables were analyzed with Student’s *t*-tests between two groups. *p* < 0.05 was considered statistically significant.

## Results

### Characteristics of the Clinical Study

We collected serum samples from 16 Chinese HIV-infected individuals. The samples were equally divided into two groups based on the detection of TB in patient lungs. The two groups had similar mean age and gender distribution (*p* > 0.05). **Table 1** shows the detailed clinical characteristics of the patients. Viral loads and mean count and proportion of CD4+ cells showed no significant differences between the two groups. Inflammation, fibrosis, and presence of opacities and cavities in chest X-ray exhibited no significant difference among the TB patients. All TB patients were confirmed by comprehensive diagnosis, such as anti-tuberculosis antibody detection, radiological changes in chest X-rays and clinical symptoms (fever, expectoration, cough, and hemoptysis). Additionally, all TB patients exhibited response to treatment. Sputum culture also provided positive evidence of Mtb infection. The group of non-TB controls of HIV-infected patients was also evaluated by assays for the detection of TB to ensure that they did not have TB.

**Table 1:** Characteristics of HIV-TB co-infected patients and HIV-noTB controls in this study.

	iTRAQ-proteomics set			ELISA set		
		
	HIV-noTB	HIV-TB	*p*	HIV-noTB	HIV-TB	*p*
Age, mean (SD)	40.3 (3.71)	42.75 (3.67)	1	41.7 (2.51)	39.9 (4.2)	1
Male (%)	50 (4)	62 (5)		48 (12)	48 (13)	
Race, Chinese (%)	100 (8)	100 (8)		100 (25)	100 (27)	
Vrial loads (10E4)	555.71	573.23	0.74	592.15	563.42	0.81
CD4+, mean (absolute)	144.63	67.57	0.33	101.76	82.57	0.47
CD4+, mean (ratio)	8.87	5.86	0.45	7.23	6.03	0.52
X-ray, symptom + (%)	0	100 (8)		0	100 (27)	
Anti-HIV + (%)	100 (8)	100 (8)		100 (26)	100 (27)	
Mtb culture + (%)	0 (0)	100 (8)		0 (0)	100 (27)	


### iTRAQ Quantification of Serum Protein Profiles

**FIGURE 2:**
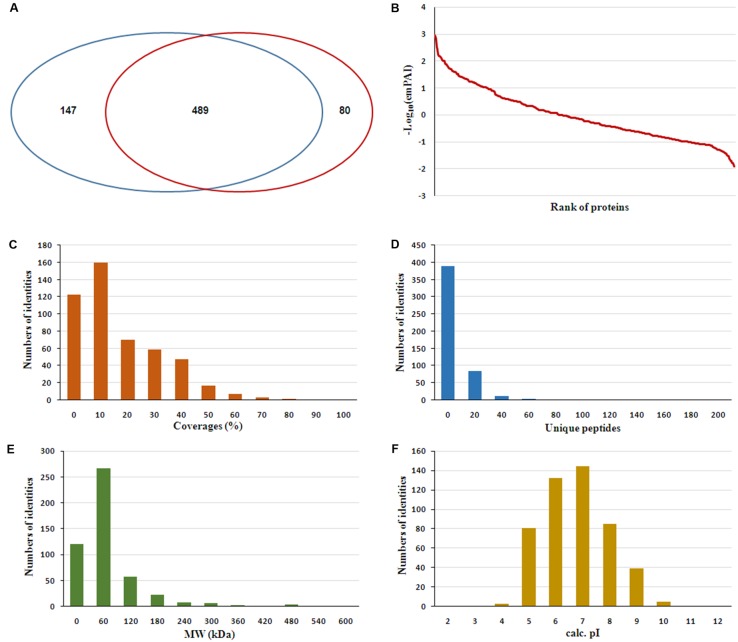
Features of the serum proteome dataset from iTRAQ shotgun analysis. **(A)** Venn diagrams displaying the number of identified proteins and the overlay of these identified proteins. **(B)** Analysis of ranges of Mascot emPAI value for protein abundance. **(C)** Distribution of proteins based on peptide coverage. **(D)** Distribution of proteins based on the number of unique peptides in the proteomics analysis. **(E)** Distribution of proteins based on molecular weight (MW). **(F)** Distribution of proteins based on pI.

**FIGURE 3:**
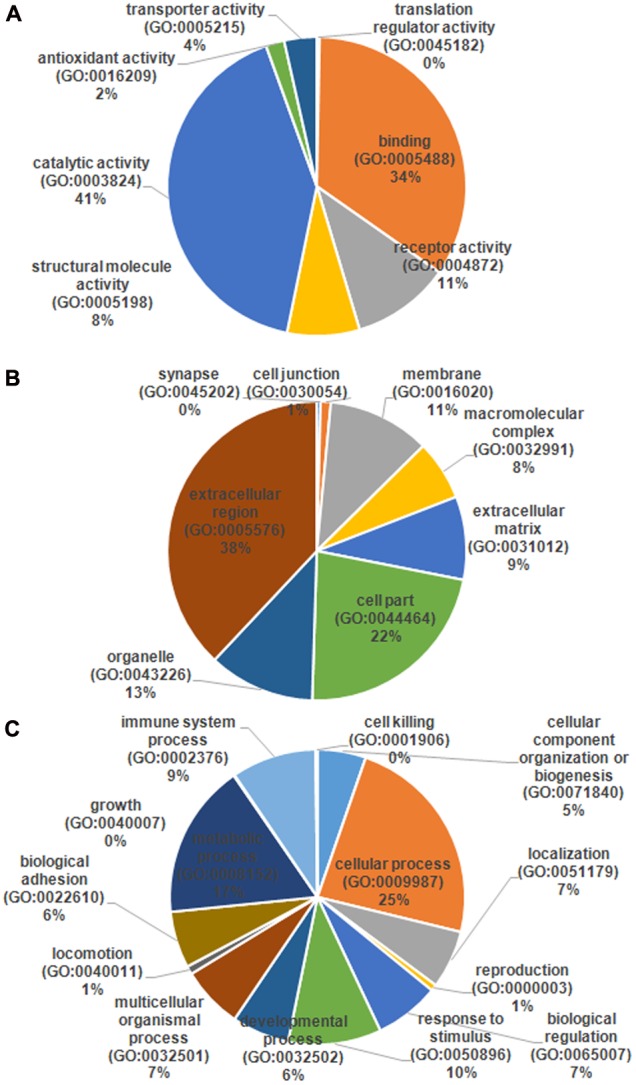
Gene ontology (GO) annotation and functional classification of identified serum proteins from all samples. GO terms for cellular compartment **(A)**, molecular function **(B)**, and biological process **(C)**.

The serum samples from the 16 HIV-noTB and HIV-TB patients were analyzed by iTRAQ. In total, 716 proteins were identified (at least one unique peptide with FDR < 0.05, **Supplementary Table S1**), including 489 proteins that were common in the two sets of iTRAQ labeling. Overall, 489 proteins identified from all 16 samples were subjected to further analysis (**Figure 2A**). The abundance of all 489 proteins was within the range of 3 to -2, as revealed by their exponentially modified protein abundance index (emPAI) (**Figure 2B**). Among the 489 identified proteins, the average coverage of peptide sequences was 17.2% (**Figure 2C**). There were 77 proteins with more than 40% coverage of peptide sequences. There were 16 proteins with more than 40 uniquely identified peptides and 83 with 20–40 uniquely identified peptides; the remaining proteins had 1–20 peptides (**Figure 2D**). Forty-five proteins were larger than 180 kDa, 57 were between 120 and 180 kDa, 266 between 60 and 120 kDa, and 121 between 0 and 60 kDa (**Figure 2E**). The protein pI values were distributed from 4 to 10; in most (361 proteins), the range was 6–8 (**Figure 2F**). The molecular functions, subcellular localizations and biological processes of the identified proteins were analyzed via GO annotation. Catalytic activity (41%), binding (34%) and receptor activity (11%) were the most highly represented categories in the identified proteins; transporter activity (4%), antioxidant activity (2%) and translation regulator (0.7%) were also readily identified (**Figure 3B**). Extracellular region (38%), cell part (22%) and membrane (11%) were the most represented classes of subcellular localization in the identified proteins (**Figure 3A**). These proteins were found to be involved primarily in the immune system processes (9%) and response to stimuli (10%) (**Figure 3C**).

### Differential Serum Proteins between HIV-TB Co-infected and HIV-Infected Patients

**FIGURE 4:**
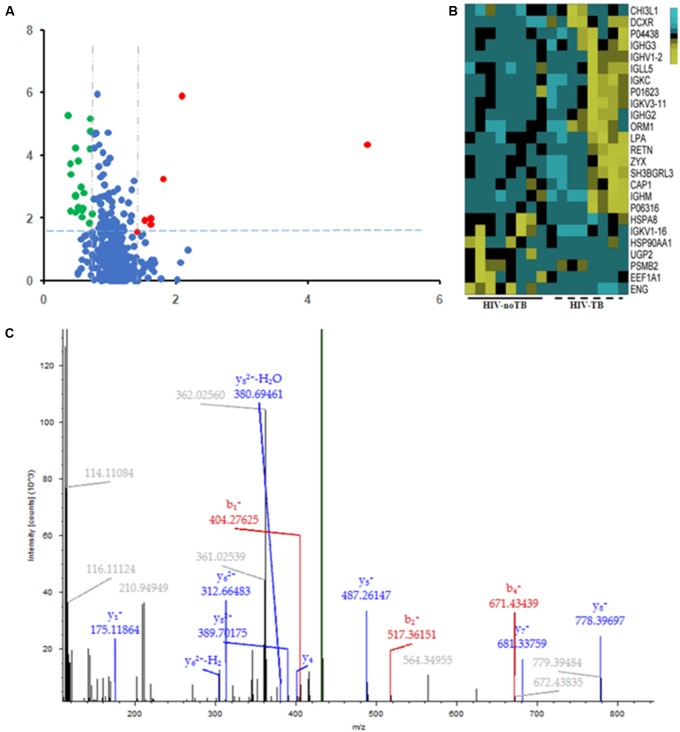
Differential expression patterns of proteins in the sera of HIV-TB co-infected patients and HIV-noTB controls. **(A)** Volcano plot of differentially expressed proteins in the two groups. 7 up-regulated proteins were colored in red, and 18 down-regulated proteins were colored in green. **(B)** Heat map illustrating the levels of 25 dysregulated serum proteins. All 25 proteins showed significant changes between the two groups (*P* < 0.05, fold change > 1.5 or < 0.37). Each group contained eight samples. **(C)** A typical MS/MS spectrum of one presentative peptide from endoglin protein.

Of the 489 proteins identified from all the samples, 7 were up-regulated (ratio HIV-TB/HIV ≥ 1.3, *p* < 0.05) and 18 were down regulated (ratio HIV-TB/HIV ≤ 0.67, *p* < 0.05) (**Figure 4** and **Table 2**). The up-regulated proteins were endoglin (ENG), UTP-glucose-1-phosphate uridylyltransferase (UGP2), elongation factor 1-alpha 1 (EEF1A1), Ig kappa chain V-I region (IGKV1-16), proteasome subunit beta type-2 (PSMB2), heat shock protein (HSP) 90-alpha (HSP90AA), and heat shock cognate 71 kDa protein (HSPA8). The down-regulated proteins were Immunoglobulin (Ig) lambda-like polypeptide 5 (IGLL5), Ig gamma-3 chain C region (IGHG3), Chitinase-3-like protein 1 (CHI3L1), adenylyl cyclase-associated protein 1 (CAP1), Ig gamma-2 chain C region (IGHG2), Ig mu chain C region (IGHM), Ig kappa chain C region (IGKC), Ig kappa chain V-III region, alpha-1-acid glycoprotein 1 (ORM1), SH3 domain-binding glutamic acid-rich-like protein 3 (SH3BGRL3), resistin (RETN), apolipoprotein (a) (LPA), Ig lambda chain V-I region BL2, Ig heavy chain V-II region, zyxin (ZYX), L-xylulose reductase (DCXR), Ig kappa chain V-III region VG (IGKV3-11), and Ig heavy chain V-I region V35 (IGHV1-2). We focused on these differentially expressed proteins for subsequent analysis.

**Table 2:** Differentially expressed proteins in the sera of HIV positive patients with active tuberculosis (TB) relative to controls (only HIV positive patients).

Accession	Gene symbol	Description	AAs	MW (kDa)	Calc. pI	Fold change	*p*
P17813	*ENG*	Endoglin	658	70.533	6.61	4.90	4.48E-05
Q16851	*UGP2*	UTP–glucose-1-phosphate uridylyltransferase	508	56.905	8.15	2.08	1.33E-06
P68104	*EEF1A1*	Elongation factor 1-alpha 1	462	50.109	9.01	1.80	5.46E-04
P04430	*IGKV1-16*	Ig kappa chain V-I region BAN	108	11.833	8.44	1.62	1.06E-02
P49721	*PSMB2*	Proteasome subunit beta type-2	201	22.822	7.02	1.62	1.55E-02
P07900	*HSP90AA1*	Heat shock protein HSP 90-alpha	732	84.607	5.02	1.52	1.24E-02
P11142	*HSPA8*	Heat shock cognate 71 kDa protein	646	70.854	5.52	1.51	3.34E-02
B9A064	*IGLL5*	Immunoglobulin lambda-like polypeptide 5	214	23.049	8.84	0.64	3.47E-02
P01860	*IGHG3*	Ig gamma-3 chain C region	377	41.26	7.9	0.61	1.53E-03
P36222	*CHI3L1*	Chitinase-3-like protein 1	383	42.598	8.46	0.61	3.75E-02
Q01518	*CAP1*	Adenylyl cyclase-associated protein 1	475	51.869	8.06	0.59	4.93E-03
P01859	*IGHG2*	Ig gamma-2 chain C region	326	35.878	7.59	0.58	5.66E-03
P01871	*IGHM*	Ig mu chain C region	452	49.276	6.77	0.58	9.05E-03
P01834	*IGKC*	Ig kappa chain C region	106	11.602	5.87	0.57	1.03E-03
P01623	*P01623*	Ig kappa chain V-III region WOL	109	11.739	8.91	0.56	4.70E-02
P02763	*ORM1*	Alpha-1-acid glycoprotein 1	201	23.497	5.02	0.53	4.82E-03
Q9H299	*SH3BGRL3*	SH3 domain-binding glutamic acid-rich-like protein 3	93	10.431	4.93	0.52	1.53E-04
Q9HD89	*RETN*	Resistin	108	11.411	6.86	0.48	1.92E-03
P08519	*LPA*	Apolipoprotein(a)	4548	500.995	5.88	0.48	6.44E-03
P06316	*P06316*	Ig lambda chain V-I region BL2	130	13.556	7.77	0.48	2.05E-03
P04438	*P04438*	Ig heavy chain V-II region SESS	147	16.312	7.09	0.48	6.15E-05
Q15942	*ZYX*	Zyxin	572	61.238	6.67	0.41	3.89E-04
Q7Z4W1	*DCXR*	L-xylulose reductase	244	25.897	8.1	0.39	1.90E-04
P04433	*IGKV3-11*	Ig kappa chain V-III region VG (Fragment)	115	12.567	4.96	0.39	6.17E-03
P23083	*IGHV1-2*	Ig heavy chain V-I region V35	117	13	9.55	0.37	5.68E-06


### GO Annotation and Functional Classification of Differentially Expressed Protein

**FIGURE 5:**
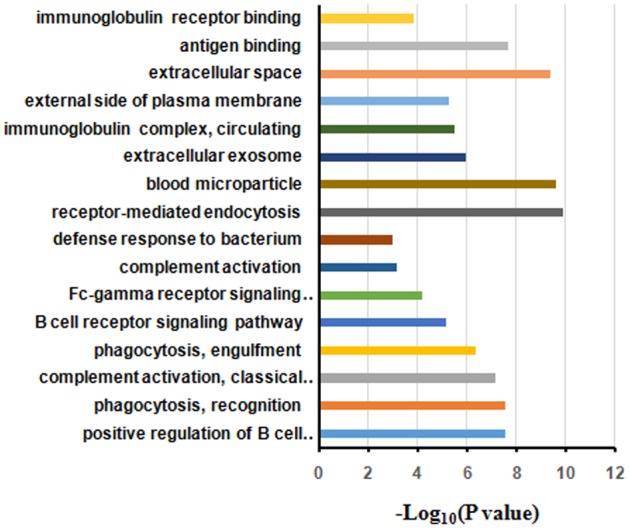
Gene ontology annotation and enrichment analysis of differentially expressed proteins in HIV-TB serum samples.

The differentially expressed proteins were subject to the DAVID webtool for GO enrichment analysis. The results showed that the differentially expressed proteins were enriched in certain cellular components, molecular functions and biological processes (**Figure 5**). According to the classification for cellular components, these proteins were enriched in blood microparticles (1.25E-10), extracellular exosomes (2.45E-10), and immunoglobulin complexes, as well as circulating proteins (1.09E-6) (**Table 3**). In terms of the molecular functions, the proteins were enriched in antigen binding (4.30E-10) and immunoglobulin receptor binding (2.27E-08) (**Table 3**). These proteins were IGHG3, IGKC, IGHM, IGLL5, IGHG2, IGHV1-2, and IGKV3-11. Most of the differential proteins were enriched in biological processes, including positive regulation of B cell activation (2.15E-08), phagocytosis, recognition (2.94E-08), complement activation (2.98E-08), phagocytosis, engulfment (7.49E-08) and B cell receptor signaling (4.46E-087) (**Table 3**). These proteins were IGHG3, IGKC, IGHM, IGLL5, IGHG2, and IGKV3-11.

**Table 3:** Significantly enriched GO terms for the differentially expressed proteins. BP, biological process; CC, cell component; MF, molecular function.

Category	GO number	GO Term	Number of proteins	%	*p*	Uniprot IDs
BP	GO:0050871	Positive regulation of B cell activation	5	23.81	2.15E-08	P01860, P01834, P01871, B9A064, P01859
BP	GO:0006910	Phagocytosis, recognition	5	23.81	2.94E-08	P01860, P01834, P01871, B9A064, P01859
BP	GO:0006958	Complement activation, classical pathway	6	28.57	2.98E-08	P01860, P01834, P01871, B9A064, P01859, P04433
BP	GO:0006911	Phagocytosis, engulfment	5	23.81	7.49E-08	P01860, P01834, P01871, B9A064, P01859
BP	GO:0050853	B cell receptor signaling pathway	5	23.81	4.46E-07	P01860, P01834, P01871, B9A064, P01859
BP	GO:0038096	Fc-gamma receptor signaling pathway involved in phagocytosis	5	23.81	6.99E-06	P01860, P01834, P07900, P01859, P04433
BP	GO:0006956	Complement activation	4	19.05	6.60E-05	P01860, P01834, P01859, P04433
BP	GO:0042742	Defense response to bacterium	4	19.05	6.99E-04	P01860, P01834, B9A064, P01859
BP	GO:0006898	Receptor-mediated endocytosis	4	19.05	9.73E-04	P01834, P07900, Q01518, P04433
CC	GO:0072562	Blood microparticle	8	38.10	1.25E-10	P02763, P01860, P01834, P11142, P01871, B9A064, P01859, P04433
CC	GO:0070062	Extracellular exosome	17	80.95	2.45E-10	Q16851, P68104, P02763, P01860, Q9H299, P01871, B9A064, Q01518, P04433, P01834, P11142, P07900, P01859, P36222, Q7Z4W1, Q9HD89, P49721
CC	GO:0042571	Immunoglobulin complex, circulating	4	19.05	1.09E-06	P01860, P01834, B9A064, P01859
CC	GO:0009897	External side of plasma membrane	6	28.57	3.15E-06	P01860, P01834, P01871, B9A064, P01859, P17813
CC	GO:0005615	Extracellular space	10	47.62	5.61E-06	P68104, P02763, P01860, P01834, P11142, P01871, P01859, P36222, P17813, Q9HD89
MF	GO:0003823	Antigen binding	7	33.33	4.30E-10	P01860, P01834, P01871, B9A064, P01859, P23083, P04433
MF	GO:0034987	Immunoglobulin receptor binding	5	23.81	2.27E-08	P01860, P01834, P01871, B9A064, P01859
MF	GO:0004252	Serine-type endopeptidase activity	5	23.81	1.35E-04	P01860, P01834, P01859, P04433, P08519


### PPI Networks of the Differentially Expressed Protein

**FIGURE 6:**
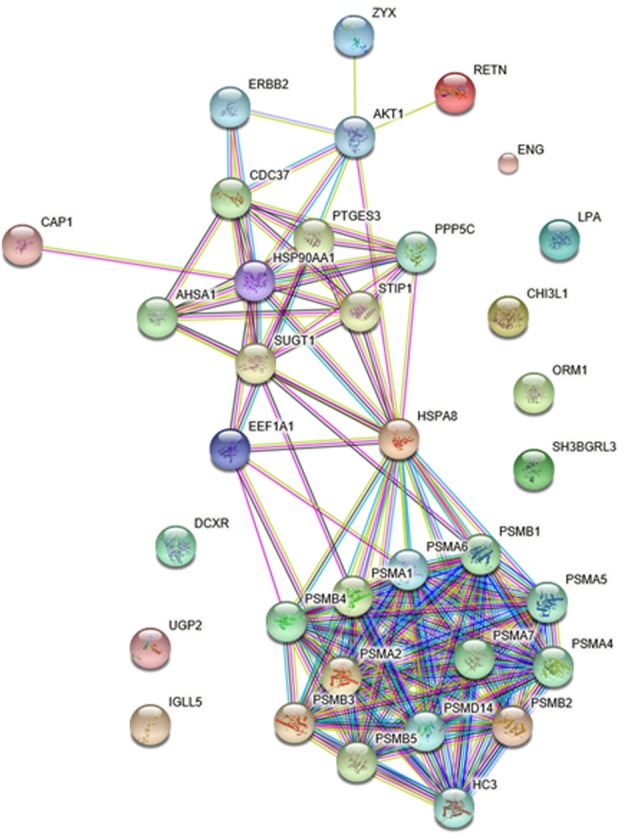
Protein–protein interaction (PPI) networks of differentially expressed proteins in HIV-TB serum samples.

To gain more insight into the biological functions and molecular characteristics of the differentially expressed proteins, PPI networks were constructed by the STRING database, which has a collection of known and predicted protein–protein interactions, including direct (physical) and indirect (functional) associations (**Figure 6**). Of the 25 differentially expressed proteins, only 15 were found in the STRING database: ENG, UGP2, EEF1A1, PSMB2, HSP90AA1, HSPA8, IGLL5, CHI3L1, CAP1, ORM1, SH3BGRL3, RETN, LPA, ZYX and DCXR (**Figure 6**). The STRING database also revealed that proteins interacted with bait proteins such as PSMB1, PSMB4 and PSMB5 in the network (**Figure 6**). All proteins in the PPI network were also subjected to GO enrichment analysis. The results showed that the proteins were enriched in biological processes such as antigen processing, presentation of exogenous peptide antigen via MHC class I, TAP-dependent pathway, and immune response-regulating cell surface receptor signaling pathway (**Table 4**).

**Table 4:** Significantly enriched GO terms in the protein–protein interaction network for the differentially expressed proteins.

GO number	GO Term	Number of proteins	*p*	Genes
GO.0002479	Antigen processing and presentation of exogenous peptide antigen via MHC class I, TAP-dependent	13	3.09E-21	HC3, PSMA1, PSMA2, PSMA4, PSMA5, PSMA6, PSMA7, PSMB1, PSMB2, PSMB3, PSMB4, PSMB5, PSMD14
GO.0002768	Immune response-regulating cell surface receptor signaling pathway	17	1.26E-18	AKT1, ERBB2, HC3, HSP90AA1, IGLL5, PSMA1, PSMA2, PSMA4, PSMA5, PSMA6, PSMA7, PSMB1, PSMB2, PSMB3, PSMB4, PSMB5, PSMD14


### Validation of iTRAQ Results

**FIGURE 7:**
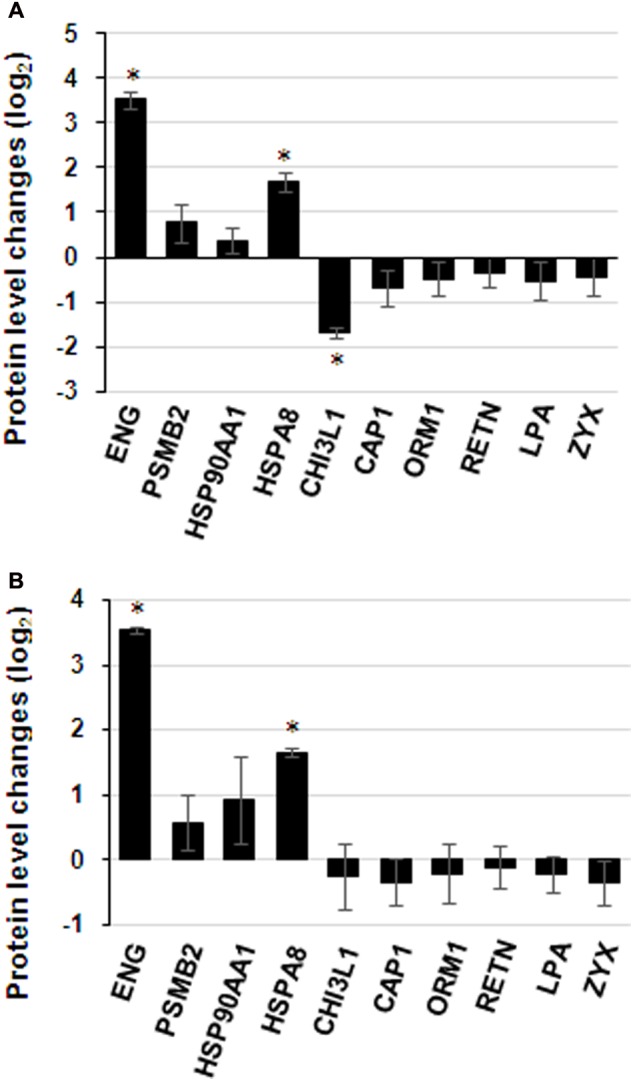
Expression patterns of selected protein candidates in the sera of HIV-TB co-infected patients and HIV-noTB controls using iTRAQ analysis and validation using ELISA assay. **(A)** Fold change of protein levels from iTRAQ analysis and validation using ELISA assay. **(B)** Fold change of protein levels using ELISA analysis after increasing the sample size. ^∗^*p* < 0.05, ^∗∗^*p* < 0.01, ^∗∗∗^*p* < 0.001 (HIV-TB serum vs. control HIV-noTB serum). The data are shown as the mean values ± SD.

Commercially available ELISA kits for the differentially expressed proteins identified by iTRAQ proteomics were obtained to validate the iTRAQ results for the 16 samples. Among the 25 differentially expressed proteins, 8 immunoglobulins were excluded from ELISA: IGKV1-16, IGLL5, IGHG3, IGHG2, IGHM, IGKC, IGKV3-11 and IGHV1-2. Only 10 proteins from remains had available commercial ELISA kits. The ELISA results were similar to those of iTRAQ, which showed statistically significant differences in the levels of proteins ENG, HSPA8 and CHI3L1 (**Figure 7A**). ENG was found to be increased by 11.5 times by ELISA in contrast to 4.9 times by iTRAQ. The other proteins showed on significant elevation or reduction by ELISA. To ascertain the clinical significance of the differentially expressed proteins as a candidate biomarker, we applied ELISA to analyze samples from more patients (25 HIV-noTB and 27 HIV-TB patients). Average values of ENG and HSPA8 exhibited the most significant differences between the HIV-TB and HIV-noTB patient sera (*p* < 0.005) (**Figure 7B**). However, the other proteins showed no significant differences at protein level.

## Discussion

At present, co-infection of Mtb and HIV is a tremendous threat for HIV patient due to the widespread distribution of Mtb and high risk of death. However, the current diagnostic methods for pulmonary TB at early stage are insufficient. With the improvements in molecular biology, diverse molecular methods have been developed to detect TB or host responses, such as PCR and proteomics. Quantitative proteomics such as iTRAQ have been applied to screen for disease markers in patient serum via the detection of TB or host responses. In the specific case of TB-HIV co-infection, mycobacterial infection can accelerate the progression of AIDS, and HIV patients are susceptible to mycobacteria. Because of the mutualism between TB and HIV infection, the presence of specific host responses may be used as potential sensitive disease markers for the detection of TB in HIV patients. Therefore, we investigated the differences in protein level in serum samples from HIV-TB co-infected patients and HIV-infected patients.

Characterization of molecular biomarker for HIV-TB co-infection in serum by proteomics has been widely explored. However, previous studies mainly focused on the populations in Western and African countries (Johnson et al., 2014; Achkar et al., 2015; Jayakumar et al., 2015). To the best of our knowledge, the survey has been limited in Chinese populations. Host responses may be quite varied in terms of different race. This is especially true since the HIV-1 B subtype is the predominant genotype in China, whereas other subtypes are more common in Western and African countries (Thomson et al., 2002; Lu et al., 2008; Su et al., 2014; Junqueira and Almeida, 2016; Ellis, 2017). We therefore believed that it is worth understanding a broad spectrum in demographics and providing the basis for adjunctive rapid TB diagnostics in HIV patients among Chinese population. In this study, approximately 500 proteins were identified in the sera by quantitative proteomics. 25 proteins were identified with statistical changes between the HIV-noTB and HIV-TB co-infection groups. This suggests that there are significant differences in host responses between in HIV-TB and HIV-noTB patients, which may be used as candidate disease markers of TB in HIV patients. Compared with previous studies, results were similar in terms of the number of differentially expressed proteins and their functions. Approximately 20 differentially expressed proteins were enriched along similar biological pathways. However, the actual differentially expressed proteins between the two studies were quite different (**Table 2**). It suggests that the host responses may be different between Chinese patients and patients of other races.

### Differentially Expressed Proteins Related to Host Response

Of the 25 differentially expressed proteins, several play biological functions related to host responses (**Table 3**). Among these host proteins, 7 candidates presented the up-regulation in HIV-TB panels. ENG (endoglin) is a major glycoprotein of the vascular endothelium and regulates angiogenesis by facilitating the binding of endothelial cells to integrins or other RGD receptors. This suggests that ENG acts as a TGF-beta coreceptor in the TGF beta/BMP signaling pathway. In other words, TGF beta signaling and angiogenesis are related to the pathogenicity of Mtb. One of the hallmarks of TB is the presence of granulomas, which are tiny clusters of immune cells locked onto bacteria. Studies have shown that Mtb can escape from these intercellular “jails” to spread throughout the body via new blood vessels recruited by angiogenesis. New blood vessels linked to granulomas provide oxygen and a route for the spreading of bacteria. The induction of ENG expression in HIV-TB co-infected patients compared to HIV-infected patients is an example of a major host response. UGP2 (UDP-glucose pyrophosphorylase 2) is an important intermediary in mammalian carbohydrate interconversions and usually plays an important role in increasing flux to glycogen synthase (Chang et al., 1996; Scheffler et al., 2016). PSMB2 (proteasome subunit beta 2) is a component of the 20S-PA28 proteasome complex involved in proteolytic degradation, which is required for the generation of a subset of major histocompatibility complex (MHC) class I-presented antigenic peptides. According to studies on the control Mtb infection, MHC class I-restricted CTLs are central players in protective immunity (Cho et al., 2000). HSP90AA1 (heat shock protein 90 alpha family class A member 1) is a molecular chaperone involved in diverse biological processes such as signal transduction through the promotion of maturation and structural maintenance of specific target proteins (Forsythe et al., 2001; Martinez-Ruiz et al., 2005;

Yang et al., 2005; Khurana and Bhattacharyya, 2015; Woodford et al., 2016). It has a function in the regulation of the transcription machinery and plays a key role in host immune response against bacteria. HSPA8 [heat shock protein family A (Hsp70) member 8] is also a molecular chaperone protein similar to HSP90AA1 with significant roles in host responses due to its binding with bacterial LPS and mediating inflammatory responses (Yahata et al., 2000; Triantafilou et al., 2001; Shang et al., 2014). So far, there has been no evidence that the levels of these proteins were significantly altered in the sera of TB patients from published proteomics data. In our study, the up-regulated proteins in HIV-TB co-infected patient sera were found to play critical roles in host responses to bacteria, especially ENG, which is involved in important processes during Mtb infection. It suggests that these proteins could be candidate disease markers for HIV-TB co-infection.

Among the down-regulated proteins identified from our proteomics analysis, IGLL5, IGHG3, CHI3L1, IGHG2, IGHM, IGKC, IGKV3-11 and IGHV1-2 are immunoglobulins, which are pivotal proteins in host responses to pathogens. Except to immunoglobulins, 7 other proteins existed for the down-regulated in the HIV-TB co-infection. Chitinase-3-like protein 1 (CHI3L1) is a mammalian glycoprotein and its expression is associated with tumor growth (Libreros et al., 2013; Chiang et al., 2015; Steponaitis et al., 2016). ORM1 (Alpha-1-acid glycoprotein 1) is a transport protein that functions in the regulation of the immune system during acute-phase reaction (Fitos et al., 2006; Zsila and Iwao, 2007). SH3 domain-binding glutamic acid-rich-like protein 3 (SH3BGRL3) is a regulator of glutaredox-in activity, which is important for antioxidant defense. RETN (Resistin) has the capacity to suppress the ability of insulin to stimulate glucose uptake into adipose cells. The relationship between insulin and TB has been studied for a long time. In the transcriptome of host peripheral blood cells of TB patients, insulin-sensitive genes were identified as differentially regulated (Lesho et al., 2011). Insulin signaling is involved in the proliferation and activation of T-cells by the regulation of the mTOR pathway (Hay and Sonenberg, 2004). However, there were some proteins among those that were down-regulated with functions not related to host responses. For example, ZYX (zyxin) is an adhesion plaque protein, which is involved in signal transduction via binding to alpha-actinin or the CRP protein. LPA (Lipoprotein) is a major component of lipoproteins with serine proteinase activity for autoproteolysis. CAP1 (Adenylyl cyclase-associated protein 1) can directly mediate filaments to regulate developmental and morphological processes.

### Candidate Disease Markers of TB in HIV-Infected Patients

Our iTRAQ proteomics experiment tested only 8 samples in each group. The limitation due to group size could result in the lack of validation of disease markers from serum proteins. Therefore, the size of the patient groups was increased for validation with ELISA assay to confirm the proteomics data. To assess the clinical significance of differentially expressed proteins as candidate biomarkers, we used ELISA assay to analyze samples from more patients (25 HIV-noTB and 27 HIV-TB patients). Given the lack of specificity of host responses to TB in HIV-infected patients, the proteins involved in common pathways in responses against pathogens could not be validated as biomarkers. Thus, these proteins showed no significant changes after we increased the sample size. From PPI network, ENG does not have any link to networks according to current knowledge including most interactions reported in the literature. It is suggested that ENG acts more independently than other proteins during host responses to pathogens.

To summary, our results showed the detection of host responses in the sera of TB-HIV co-infected patients using protein levels. Furthermore, in contrast with previous studies, we focused on Chinese patients, which revealed a distinct group of differentially expressed proteins compared with other races. Although, the current study was limited by the relative small sample size, our results were able to demonstrate statistically significant differences for TB detection between HIV-TB co-infected individuals and the controls. Moreover, our results were convinced by a highly reproducible data. Further studies using larger sample sizes in Chinese populations from both HIV-noTB and HIV-TB subjects are warranted to validate the robustness and potential clinical value of our identified TB biomarker. In a word, the candidate biomarkers identified from these differentially expressed proteins provide the basis for rapid clinical diagnosis of TB in Chinese HIV-infected individuals.

## Author Contributions

CC, TY, and LL collected the data and wrote the draft. QJ and JW designed the project and reviewed the manuscript.

## Conflict of Interest Statement

The authors declare that the research was conducted in the absence of any commercial or financial relationships that could be construed as a potential conflict of interest.

## Footnotes

1 http://www.uniprot.org/uniprot

2 http://david.abcc.ncifcrf.gov/

3 http://string-db.org/cgi/

## Supplementary Material

**TABLE S1 |** All information for proteins identified by proteomics.
